# Species-specific differences in relative eye size are related to patterns of edge avoidance in an Amazonian rainforest bird community

**DOI:** 10.1002/ece3.1194

**Published:** 2014-09-08

**Authors:** Cristina Martínez-Ortega, Eduardo SA Santos, Diego Gil

**Affiliations:** 1 Departamento de Ecología Evolutiva, Museo Nacional de Ciencias Naturales (CSIC)José Gutiérrez Abascal 2, 28006, Madrid, Spain; 2 Departamento de Ecologia, Instituto de Biociências, Universidade de São PauloRua do Matão, trav. 14, no 321, Cidade Universitária, 05508-090, São Paulo, SP, Brazil

**Keywords:** Avian vision, ecology, habitat use, light environment, perception

## Abstract

Eye size shows a large degree of variation among species, even after correcting for body size. In birds, relatively larger eyes have been linked to predation risk, capture of mobile prey, and nocturnal habits. Relatively larger eyes enhance visual acuity and also allow birds to forage and communicate in low-light situations. Complex habitats such as tropical rain forests provide a mosaic of diverse lighting conditions, including differences among forest strata and at different distances from the forest edge. We examined in an Amazonian forest bird community whether microhabitat occupancy (defined by edge avoidance and forest stratum) was a predictor of relative eye size. We found that relative eye size increased with edge avoidance, but did not differ according to forest stratum. Nevertheless, the relationship between edge avoidance and relative eye size showed a nonsignificant positive trend for species that inhabit lower forest strata. Our analysis shows that birds that avoid forest edges have larger eyes than those living in lighter parts. We expect that this adaptation may allow birds to increase their active daily period in dim areas of the forest. The pattern that we found raises the question of what factors may limit the evolution of large eyes.

## Introduction

Most vertebrates rely on light for foraging, communication, and predator avoidance, and numerous species adjust their daily routines as a function of available light (Thomas et al. 2002; Berg et al. 2006). But habitats vary widely in the amount of light that they are exposed to (Endler 1993). Several adaptations have been shown to allow organisms to survive in different ambient light conditions (McNab 2002), including an increase in relative eye size in habitats where light is scarce (Warrant 2004). This pattern has been found in diverse vertebrate orders, from tarsiers and humans to abyssal fish (Warrant 2004; Kirk 2006; Pearce and Dunbar 2012). Anatomical data show that larger eyes can accommodate larger pupillae and corneas, more photoreceptors that allow increased visual acuity, a larger visual field width, and thus the possibility of seeing in dim light conditions (Martin and Katzir 2000; Veilleux and Lewis 2011). In birds, species with relatively larger eyes have been shown to be more likely to feed on mobile prey and have nocturnal habits (Garamszegi et al. 2002), flee at a longer distance from predators (Møller and Erritzøe 2010, 2014) and sing earlier at dawn (Thomas et al. 2002; Berg et al. 2006). Additionally, a modification in eye shape caused by an increase in axial depth with respect to the corneal diameter has been found in nocturnal birds, although this pattern has not been verified in a comparative analysis correcting for phylogeny (Hall and Ross 2007).

**Figure 1:**
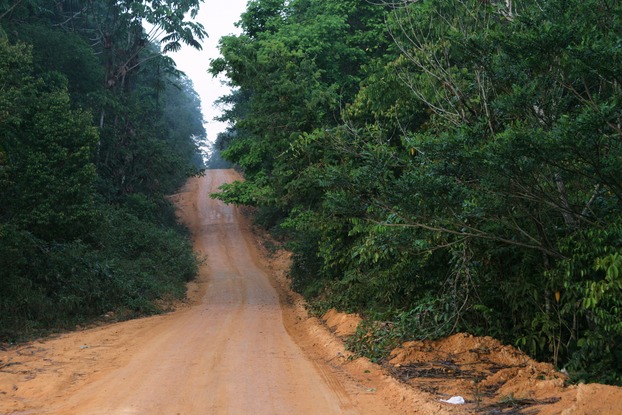
Forest edge near Manaus (Amazonas, Brazil). Forest avian species differ in the degree to which they avoid or favor forest edges and can thus be classified along a continuum of edge avoidance.

In structurally complex forests, there are large differences in light levels between strata and at different distances from the edge (Endler 1993), favoring the evolution of fine adaptations in communication strategies (Endler and Thery 1996). For instance, bird species that live in dark forest areas have highly conspicuous plumage patterns, which are expected to be advantageous in intraspecific communication (Marchetti 1993; Shultz and Burns 2013). However, we know of no specific test linking relative eye size with habitat darkness in such a structurally complex environment. We predicted that relative eye size should be dependent on within-forest microhabitat occupancy (Fig.1). We tested our hypothesis in a species-rich rainforest bird community in the Amazonas Central Region (Cohn-Haft et al. 1997). In this habitat, strong differences among species in microhabitat usage allow a fine two-dimensional separation in distance to the edge and forest stratum (Stotz et al. 1996). We expected eye size to increase with increasing distance to the forest edge and also to be larger for understory than for canopy birds.

## Material and Methods

### Study area and field data collection

We conducted our study in the Adolpho Ducke Forest Reserve (25 km NW of Manaus, Brazil) in October 2009, which corresponds to the peak of the breeding season in this area (Stouffer et al. 2013). This is a large (10,000 ha) homogenous stretch of *terra firme* tropical forest with a continuous canopy around 37 m in height (Cohn-Haft et al. 1997). We selected an area of 900 by 300 m, running along the southern edge of the forest. In this area, we established three parallel paths at 100, 200, and 300 m from the forest edge. Each transect was further divided into 100 m stretches, creating a grid of 27 sound recording points. We recorded dawn chorus at these points (continuously between 05.00 and 09.00 am; 48 kHz, 16 bits) using three automatic “Song-Meter 1” units (Wildlife Acoustics) during 9 days, all three transects being sampled each day at a different point. Recordings were divided in 5-min intervals and birds identified as present/absent in each interval by a bird expert (Marconi Campos-Cerqueira, INPA, Brazil). All species could be identified with certainty, except *Thraupis palmarum* and *T. episcopus*, which have similar songs. Given their similar ecology and morphology, we arbitrarily assigned all recordings of this genus to *T. palmarum*.

A total of 136 bird species from 30 families were detected in the 108 h of recording time (see Appendix 1). We arbitrarily selected species that had been detected in more than half of the days (≥6 detection, *N* = 66 species) to avoid introducing noise from uncommon species into the analyses. We calculated an edge-avoidance index by dividing the number of days a species was detected in the innermost transect by the days the bird had been detected in all transects.

We tested the internal reliability of our edge-avoidance index by dividing the sample in two half-samples (the first 5 days against the last 4 days) and comparing the scores, which were found to be repeatable (Pearson's *r *=* *0.44, *N* = 40, *P *<* *0.01; sample is smaller because not all 66 species were detected in both half-samples). Although our method does not take into account imperfect detection (MacKenzie et al. 2004), we checked its reliability by testing the relationship between our edge-avoidance index and a published classification of edge species (Cohn-Haft et al. 1997). We found that birds that favor edges according to Cohn-Haft et al. (1997) had a lower edge-avoidance index than those who do not favor edges (PGLS: estimate (SE) = −0.14 (0.06), *F*_2,64_ = 5.01, *P *<* *0.01), suggesting that our edge-avoidance index is a robust proxy of habitat preference in terms of edge versus. forest interior.

Data on preference for vegetation strata were obtained from a published source (Stotz et al. 1996) and was defined in three categories: understory (terrestrial +  understory), middle height, and canopy. Body size was taken as the midpoint in a range of body lengths from a common data source (del Hoyo et al. 1992–2001).

### Eye size measurements

For a subsample of species (*N* = 42), direct eye size measurements from dissected specimens conserved in ethanol were available in Ritland's (1982) monograph, and we averaged values for all samples that were provided (mean number of samples = 1.5, SD = 1.04). We estimated eye volume assuming the shape of the eye to correspond to an oblate spheroid (Garamszegi et al. 2002), using the equation:
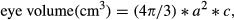
where *a* is the equatorial radius (TM1/2 in Ritland's) and *c* is the polar radius (TM2/2 in Ritland's), measured in cm.

For the remaining species (*N* = 24), we obtained eye size estimates by measuring exposed eye area in a sample of photographs obtained from different Internet sources (mean number of pictures per species = 2.86, SD = 0.34). Briefly, photographs were scaled on average bill measurements and the exposed eye area measured with the “polygon” tool in the software ImageJ (Wayne Rasband, NIH, USA). To this end, bill data were obtained by one of us (ESAS) from stuffed birds in the Museu de Zoologia da Universidade de São Paulo (mean number of specimens per species = 2.76, SD = 0.5). Eye area was averaged over two estimates obtained by photographs using beak length and beak height as scaling parameters, respectively.

Before pooling our measurements with those from Ritland (1982), we used a linear regression to correct for differences in measurement technique. To this end, a sample of 22 species available in Ritland's was also measured in photographs. The result of this linear regression suggests that exposed eye area measured in photographs is a close estimate of eye volume as measured in dissected specimens (area (mm^2^) = eye volume (cm^3^) * 0.034 − 0.105; *F*_(1,20) _= 94.25, *P *<* *0.001, *R*^2 ^= 0.81).

Although it would have been interesting to add to our study information on axial depth, and thus, eye shape (Hall and Ross 2007), we could only obtain these data in the subsample of species studied by Ritland (1982). Additionally, given that axial diameter is very strongly associated with eye volume as calculated from transverse radii (linear regression on logs: *F*_(1,41) _= 1517.9, *P *<* *0.001, *R*^2 ^= 0.97; *β* (SE) = 1.01 (0.34)), it would seems highly unlikely to find an allometric modification of shape in these species.

### Statistical analysis

Data were analyzed with a phylogenetic linear model using packages *caper* and *ape* in R (Orme 2012; R Development Core Team 2013). We analyzed the relationship between relative eye size and edge avoidance and stratum with maximum likelihood estimates of Pagel's lambda values. We obtained a random sample of 1,000 phylogenetic trees from Jetz et al. (2012; birdtree.org), using the sampling tool available on the website. A majority-rule consensus tree is presented in Appendix 3 for illustration purposes. We repeated each model with each of the 1000 trees and report the mean slope of the phylogenetic regression and the mean two-tailed *P*-values. Model residuals did not depart from normality and homoscedasticity.

## Results

**Figure 2:**
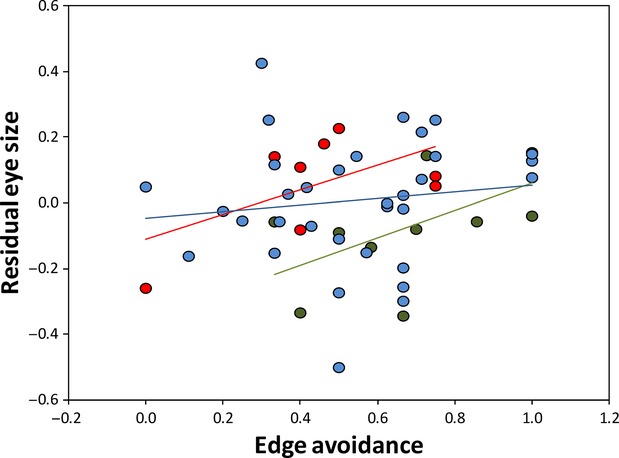
Plots showing the relationship between residual eye volume (corrected for body size) and our measure of edge avoidance for canopy (blue marks), middle stratum (green) and understory (red) birds. Data points are residuals from a regression of eye volume on body size and thus are not phylogenetically corrected. Regression lines for illustration only, slopes from the model are as follows: understory: 0.32 (0.12); medium stratum: 0.17 (0.08); and canopy: 0.12 (0.06). Slope comparisons, all *Z *<* *1.4, *P *>* *0.08.

Eye size evolution was better explained (lowest AIC) by a Brownian model (AIC = −32.10) than by an Ornstein–Uhlenbeck model (AIC = −14.15). When considering the relationship between eye size and body size, edge avoidance and forest stratum, the model with an absolute lower AIC (−51.2) included all terms and a nonsignificant interaction between edge avoidance and stratum (Table1). However, a simpler model not including the interaction showed only a slightly higher AIC (−49.70), suggesting that both models are equally parsimonious. In summary, birds had increasing relative eye sizes with increasing edge avoidance (Fig.2), and this pattern was similar for inhabitants of the three strata. Despite the nonsignificant interaction between these predictors, a comparison of slopes suggests a trend for a flatter slope in the case of canopy birds with respect to other strata (Fig.2), which goes in the direction of our a priori expectation. The phylogenetic signal of eye size in the model was strong (mean ML estimation: *λ *= 0.92).

**Table 1:** Parameter estimates (and SEs) for the best phylogenetic generalized linear model (PGLS) for eye volume, as determined from AIC comparison (see main text). Data show mean estimates for a sample of 1000 different trees. Statistics for the full model are as follows: *F*_5,61_ = 41.26, *P *<* *0.001.

Terms	Estimate (SE)	*t*	*P*
(Intercept)	−2.45 (0.26)	−9.42	<0.001
Body mass (log)	1.56 (0.13)	11.61	<0.001
Forest stratum	0.07 (0.06)	1.27	0.21
Edge avoidance	0.60 (0.23)	2.59	0.01
Edge avoidance^*^Forest stratum	−0.17 (0.09)	−1.97	0.053

## Discussion

We found that relative eye size was predicted by some microhabitat characteristics in a group of Amazonian forest birds. Birds that dwell in deeper, darker parts of the forest, furthest from the forest edge, had larger eyes for their size than birds that tend to occur in forest edges. Surprisingly, we did not find differences in eye size between birds favoring different forest strata, despite there being large differences in light conditions (Endler 1993). The relationship between edge avoidance and eye size was similar for birds inhabiting the three different strata, although the interaction showed a nonsignificant trend for a weaker relationship in the case of canopy birds. The general pattern that we found is similar to a previous study in mammals, where similar differences in eye size were found between habitat types, but not in relation to forest strata (Veilleux and Lewis 2011).

Our results provide an additional layer of variation to previous research showing that relative large eye size in birds is an adaptation to capture of moving prey, nocturnal habits, and susceptibility to predation (Garamszegi et al. 2002). Physiological evidence shows that larger eyes provide higher visual acuity through a higher number of photoreceptors, and also an absolute increase in photo-stimulation which reduces the stimulation threshold (Martin 1993; Güntürkün 1999). Bird species with relatively larger eyes start singing earlier, probably being able to forage earlier than other species (Thomas et al. 2002, 2006; Berg et al. 2006). We would expect thus larger eyes to allow extended or earlier foraging time in dwellers of forest interiors, although no present study to our knowledge has examined this possibility in this group of species.

A previous study (Møller and Erritzøe 2010) did not find differences in relative eye size between birds living in open and close European habitats, suggesting that the differences that we found may be specific of extremely dark forests such as those found in the tropics. However, we do not know whether larger eyes fully compensate for differences in ambient light, or if this compensation is only partial.

If big eyes are important for early predator detection (Møller and Erritzøe 2010, 2014) and increase the range of light conditions under which birds can forage and communicate, why do some birds have relatively small eyes? The positive relationship between relative eye and brain size has been interpreted as a suggestion that neural costs may constraint the advantage of big eyes (Garamszegi et al. 2002). However, an excess of light is detrimental for the retina cells, primarily by photo-chemical damages induced by ultraviolet and blue radiation (Marshall 1991). Indeed, some birds have evolved special anatomical structures (i.e., feathered eyelids) to shade the eyes from an excess of light (Martin and Katzir 2000). Thus, the evolution of big eyes may also be constrained by costs due to photo-chemical injury in species which are exposed to high levels of sunlight.

Edge avoidance is a highly species-specific trait that organizes the distribution of species in many forested areas (Lindell et al. 2007). Under the current scenario of habitat destruction, differences in edge avoidance may result in heterogeneous responses to habitat fragmentation, leading to species-specific patterns of resilience (Laurance et al. 2004). We expect edge avoiders to be particularly vulnerable to habitat fragmentation. Our data provide evidence that behavioral differences and microhabitat occupancy are related to morphological differences among species possibly due to patterns of physiological adaptation.

## Data accessibility

Data are available as Appendices 1 and 2 at the end of this article. Recordings are deposited at the Fonoteca Zoologica (MNCN-CSIC).

## 1 Appendix

Full list of avian species identified in the recordings, with number of detections and number of days in which each species was detected per transect. Total detections are the absolute number of 5-min intervals in which a species was detected during the duration of the study.

**Table:**

Family	Species	Total detections	Days detected in each transect		
Inner	Medium	External
TINAMIDAE	*Crypturellus soui*	1	1	0	0
TINAMIDAE	*Crypturellus variegatus*	4	3	0	0
TINAMIDAE	*Tinamus major*	3	2	1	0
ACCIPITRIDAE	*Leucopternis melanops*	2	1	0	0
ACCIPITRIDAE	*Buteo magnirostris*	20	3	3	4
FALCONIDAE	*Falco rufigularis*	2	0	1	1
RALLIDAE	*Laterallus viridis*	2	1	0	0
COLUMBIDAE	*Patagioenas plumbea*	21	3	3	0
COLUMBIDAE	Patagioenas sp.	1	1	0	0
PSITTACIDAE	*Amazona autumnalis*	177	9	9	9
PSITTACIDAE	*Amazona farinosa*	3	2	0	0
PSITTACIDAE	Amazona sp.	1	0	1	0
PSITTACIDAE	Ara sp.	1	1	0	0
PSITTACIDAE	*Brotogeris chrysoptera*	9	3	3	0
PSITTACIDAE	*Orthopsittaca manilata*	1	1	0	0
PSITTACIDAE	*Pionus menstruus*	52	7	6	9
PSITTACIDAE	*Pionus fuscus*	41	6	2	0
PSITTACIDAE	Pionus sp.	1	1	0	0
PSITTACIDAE	*Pyrilia caica*	38	5	2	1
CUCULIDAE	*Dromococcyx pavoninus*	1	0	1	0
CUCULIDAE	*Piaya cayana*	18	4	2	1
CUCULIDAE	*Piaya melanogaster*	1	0	1	0
CUCULIDAE	Piaya sp.	4	2	2	0
CAPRIMULGIDAE	*Lurocalis semitorquatus*	3	2	0	0
NYCTIBIDAE	*Nyctibius aethereus*	1	1	0	0
TROCHILIDAE	*Phaethornis ruber*	5	0	4	1
TROGONIDAE	*Trogon melanurus*	3	1	0	0
TROGONIDAE	Trogon sp.	5	3	2	0
TROGONIDAE	*Trogon viridis*	35	6	1	1
GALBULIDAE	*Galbula albirostris*	13	6	0	1
GALBULIDAE	*Galbula dea*	44	6	2	1
GALBULIDAE	*Jacamerops aureus*	5	1	2	1
BUCCONIDAE	*Bucco tamatia*	2	2	0	0
BUCCONIDAE	*Chelidoptera tenebrosa*	1	1	0	0
BUCCONIDAE	*Monasa atra*	13	5	1	1
BUCCONIDAE	*Notharchus macrorhynchos*	1	1	0	0
CAPITONIDAE	*Capito niger*	1	0	1	0
RAMPHASTIDAE	Pteroglossus sp.	2	2	0	0
RAMPHASTIDAE	*Pteroglossus viridis*	5	1	1	3
RAMPHASTIDAE	*Ramphastos tucanus*	35	7	7	5
RAMPHASTIDAE	*Ramphastos vitellinus*	5	2	1	0
RAMPHASTIDAE	*Ramphocaenus melanurus*	4	3	1	0
RAMPHASTIDAE	*Sclerurus caudacutus*	1	1	0	0
RAMPHASTIDAE	*Selenidera piperivora*	22	5	2	0
PICIDAE	*Celeus torquatus*	1	0	1	0
PICIDAE	*Melanerpes cruentatus*	2	1	0	0
PICIDAE	*Piculus chrysochloros*	2	1	0	0
PICIDAE	*Piculus flavigula*	12	4	2	0
PICIDAE	*Veniliornis cassini*	2	2	0	0
DENDROCOLAPTIDAE	*Campylorhamphus procurvoides*	1	1	0	0
DENDROCOLAPTIDAE	*Deconychura stictolaema*	3	2	0	0
DENDROCOLAPTIDAE	*Dendrexetastes rufigula*	5	2	0	0
DENDROCOLAPTIDAE	*Dendrocincla fuliginosa*	27	7	5	0
DENDROCOLAPTIDAE	*Dendrocolaptes certhia*	2	1	1	0
DENDROCOLAPTIDAE	*Dendrocolaptes picumnus*	7	4	0	0
DENDROCOLAPTIDAE	*Glyphorynchus spirurus*	7	2	3	0
DENDROCOLAPTIDAE	*Lepidocolaptes albolineatus*	2	1	0	0
DENDROCOLAPTIDAE	*Sittasomus griseicapillus*	13	2	1	0
DENDROCOLAPTIDAE	*Xiphorhynchus pardalotus*	36	7	3	0
THAMNOPHILIDAE	*Cercomacra cinerascens*	1	1	0	0
THAMNOPHILIDAE	*Cymbilaimus lineatus*	3	2	0	0
THAMNOPHILIDAE	*Frederickena viridis*	1	1	0	0
THAMNOPHILIDAE	*Gymnopithys rufigula*	25	3	1	0
THAMNOPHILIDAE	*Hypocnemis cantator*	6	1	1	0
THAMNOPHILIDAE	*Myrmeciza ferruginea*	9	2	2	1
THAMNOPHILIDAE	*Myrmotherula brachyura*	9	2	1	0
THAMNOPHILIDAE	*Myrmotherula gutturalis*	4	1	0	0
THAMNOPHILIDAE	*Myrmotherula axillaris*	1	1	0	0
THAMNOPHILIDAE	Myrmotherula sp.	1	0	1	0
THAMNOPHILIDAE	*Percnostola rufifrons*	65	6	4	3
THAMNOPHILIDAE	*Pithys albifrons*	5	2	1	1
THAMNOPHILIDAE	*Schistocichla leucostigma*	24	2	2	1
THAMNOPHILIDAE	*Thamnomanes ardesiacus*	6	1	1	0
THAMNOPHILIDAE	*Thamnomanes caesius*	2	2	0	0
THAMNOPHILIDAE	*Thamnophilus murinus*	40	8	2	1
FORMICARIIDAE	*Formicarius colma*	29	6	2	0
FORMICARIIDAE	*Herpsilochmus dorsimaculatus*	5	3	0	0
TYRANNIDAE	*Attila spadiceus*	13	2	0	0
TYRANNIDAE	*Camptostoma obsoletum*	2	0	1	1
TYRANNIDAE	*Conopias parva*	4	2	0	0
TYRANNIDAE	*Conopophaga aurita*	5	2	0	0
TYRANNIDAE	*Hemitriccus zosterops*	3	2	0	1
TYRANNIDAE	*Legatus leucophaius*	1	0	0	1
TYRANNIDAE	*lophotriccus vitiosus*	1	0	1	0
TYRANNIDAE	*Megarynchus pitangua*	10	0	1	2
TYRANNIDAE	*Myiopagis gaimardii*	29	4	1	1
TYRANNIDAE	*Myiornis ecaudatus*	15	4	1	1
TYRANNIDAE	*Myiozetetes cayanensis*	13	1	4	4
TYRANNIDAE	*Pitangus sulphuratus*	5	1	1	1
TYRANNIDAE	*Platyrinchus coronatus*	7	3	0	0
TYRANNIDAE	*Platyrinchus platyrhynchos*	2	1	0	0
TYRANNIDAE	*Rhytipterna simplex*	9	3	0	0
TYRANNIDAE	*Terenotriccus erythrurus*	2	1	0	0
TYRANNIDAE	*Todirostrum maculatum*	4	2	1	1
TYRANNIDAE	*Todirostrum pictum*	77	9	7	2
TYRANNIDAE	Todirostrum sp.	1	0	0	1
TYRANNIDAE	*Tolmomya poliocephalus*	1	1	0	0
TYRANNIDAE	*Tolmomyias assimilis*	21	4	1	1
TYRANNIDAE	*Tolmomyias poliocephalus*	52	6	5	3
TYRANNIDAE	*Tyrannus melancholicus*	5	2	0	0
TYRANNIDAE	*Tyranopsis sulphurea*	1	1	0	0
TYRANNIDAE	*Zimmerius gracilipes*	20	2	4	2
PIPRIDAE	*Pipra erythrocephala*	10	2	0	0
PIPRIDAE	*Piprites chloris*	1	1	0	0
PIPRIDAE	*Tyranneutes virescens*	1	1	0	0
COTINGIDAE	*Iodopleura fusca*	1	1	0	0
COTINGIDAE	*Lipaugus vociferans*	11	5	0	0
TROGLODYTIDAE	*Microcerculus bambla*	6	1	1	1
TROGLODYTIDAE	*Pheugopedius coraya*	5	1	1	0
TROGLODYTIDAE	*Troglodytes musculus*	13	0	1	2
TURDIDAE	*Turdus albicollis*	5	2	0	0
TURDIDAE	*Turdus ignobilis*	1	1	0	0
TURDIDAE	*Turdus leucomelas*	4	3	0	0
TURDIDAE	Turdus sp.	2	1	1	0
POLIOPTILIDAE	*Microbates collaris*	3	2	0	0
EMBERIZIDAE	*Arremon taciturnus*	4	2	1	0
CARDINALIDAE	*Caryothraustes canadensis*	2	1	1	0
CARDINALIDAE	*Saltator grossus*	30	5	2	1
CARDINALIDAE	*Saltator maximus*	2	0	0	1
THRAUPIDAE	*Chlorophanes spiza*	2	1	1	0
THRAUPIDAE	*Euphonia cayannensis*	1	1	0	0
THRAUPIDAE	*Euphonia chrysopasta*	5	3	1	0
THRAUPIDAE	*Lamprospiza melanoleuca*	1	0	0	1
THRAUPIDAE	*Tachyphonus cristatus*	2	1	0	0
THRAUPIDAE	*Tachyphonus surinamus*	21	3	3	3
THRAUPIDAE	Tangara sp.	1	1	0	0
THRAUPIDAE	*Tangara varia*	8	3	3	0
THRAUPIDAE	Thraupis sp.	31	2	1	3
VIREONIDAE	*Cyclarhis gujanensis*	5	3	1	0
VIREONIDAE	*Hylophilus muscicapinus*	66	5	4	3
VIREONIDAE	*Vireo olivaceus*	1	0	0	1
VIREONIDAE	*Vireolanius leucotis*	27	6	3	2
ICTERIDAE	*Cacicus cela*	6	1	3	1
ICTERIDAE	*Cacicus haemorrhous*	246	9	9	8
ICTERIDAE	*Celeus undatus*	3	1	0	0
ICTERIDAE	*Psarocolius viridis*	1	1	0	0

## 2 Appendix

Database used in the main analysis of the study, including index of edge avoidance calculated from distribution of days detected, body size (log converted), forest stratum, and eye volume (log converted). Please see Methods for details.

**Table:**

Species	Edge avoidance	Body size (log)	Forest stratum	Eye volume (log)
*Amazona autumnalis*	0.333	1.525	3	0.352
*Attila spadiceus*	1.000	1.284	3	0.040
*Brotogeris chrysoptera*	0.500	1.204	3	−0.123
*Buteo magnirostris*	0.300	1.568	3	0.723
*Cacicus cela*	0.200	1.407	3	0.041
*Cacicus haemorrhous*	0.346	1.433	3	0.047
*Conopophaga aurita*	1.000	1.070	1	−0.230
*Cyclarhis gujanensis*	0.750	1.176	3	−0.219
*Dendrexetastes rufigula*	1.000	1.394	2	−0.150
*Dendrocincla fuliginosa*	0.583	1.317	2	−0.197
*Dendrocolaptes picumnus*	1.000	1.435	2	0.067
*Euphonia chrysopasta*	0.750	0.978	3	−0.574
*Formicarius colma*	0.750	1.255	1	−0.099
*Galbula albirostris*	0.857	1.290	2	−0.158
*Galbula dea*	0.667	1.473	3	−0.137
*Glyphorynchus spirurus*	0.400	1.161	2	−0.619
*Gymnopithys rufigula*	0.750	1.079	1	−0.321
*Herpsilochmus dorsimaculatus*	1.000	1.061	3	−0.150
*Hylophilus muscicapinus*	0.417	1.070	3	−0.368
*Hypocnemis cantator*	0.500	1.061	2	−0.519
*Jacamerops aureus*	0.250	1.439	3	0.120
*Lipaugus vociferans*	1.000	1.415	3	0.206
*Megarynchus pitangua*	0.000	1.357	3	0.044
*Microcerculus bambla*	0.333	1.061	1	−0.287
*Monasa atra*	0.714	1.431	3	0.317
*Myiopagis gaimardii*	0.667	1.088	3	−0.646
*Myiornis ecaudatus*	0.667	0.813	3	−0.522
*Myiozetetes cayanensis*	0.111	1.237	3	−0.339
*Myrmeciza ferruginea*	0.400	1.161	1	−0.367
*Myrmotherula brachyura*	0.667	0.889	3	−0.692
*Patagioenas plumbea*	0.500	1.531	3	−0.257
*Percnostola rufifrons*	0.462	1.154	1	−0.115
*Phaethornis ruber*	0.000	0.929	1	−1.149
*Thryothorus coraya*	0.500	1.161	1	−0.392
*Piaya cayana*	0.571	1.663	3	0.281
*Piculus flavigula*	0.667	1.290	3	−0.299
*Pionus fuscus*	0.750	1.407	3	0.318
*Pionus menstruus*	0.318	1.415	3	0.331
*Pipra erythrocephala*	1.000	0.929	2	−0.488
*Pitangus sulphuratus*	0.333	1.342	2	−0.049
*Pithys albifrons*	0.500	1.079	1	−0.458
*Platyrinchus coronatus*	1.000	0.942	2	−0.445
*Pteroglossus viridis*	0.200	1.538	3	0.318
*Pyrilia caica*	0.625	1.362	3	0.000
*Ramphastos tucanus*	0.368	1.744	3	0.576
*Ramphastos vitellinus*	0.667	1.708	3	0.523
*Rhytipterna simplex*	1.000	1.301	3	−0.008
*Saltator grossus*	0.625	1.296	3	−0.104
*Schistocichla leucostigma*	0.400	1.176	1	−0.155
*Selenidera piperivora*	0.714	1.531	3	0.317
*Sittasomus griseicapillus*	0.667	1.211	2	−0.558
*Tachyphonus surinamus*	0.333	1.204	2	−0.281
*Tangara varia*	0.500	1.041	3	−0.730
*Thamnomanes ardesiacus*	0.500	1.130	1	−0.102
*Thamnophilus murinus*	0.727	1.130	2	−0.185
Thraupis sp.	0.333	1.217	3	−0.357
*Todirostrum pictum*	0.500	0.982	3	−0.651
*Tolmomyias assimilis*	0.667	1.122	3	−0.318
*Tolmomyias poliocephalus*	0.429	1.079	3	−0.473
*Troglodytes aedon*	0.000	1.079	1	−0.662
*Trogon viridis*	0.750	1.423	3	0.232
*Turdus albicollis*	1.000	1.366	2	0.020
*Tyrannus melancholicus*	1.000	1.326	3	−0.110
*Vireolanius leucotis*	0.545	1.161	3	−0.143
*Xiphorhynchus pardalotus*	0.700	1.352	2	−0.092
*Zimmerius gracilipes*	0.250	1.021	3	−0.540

## 3 Appendix

Hypothetic phylogenetic reconstruction (consensus tree following majority rules) of the species used in the study, derived from birdtree.org. Note that in the analysis, 1000 random trees were used.
